# Neural correlates of saccadic inhibition in healthy elderly and patients with amnestic mild cognitive impairment

**DOI:** 10.3389/fpsyg.2013.00467

**Published:** 2013-07-24

**Authors:** K. K. Alichniewicz, F. Brunner, H. H. Klünemann, M. W. Greenlee

**Affiliations:** ^1^Institute of Experimental Psychology, University of RegensburgRegensburg, Germany; ^2^Departments of Psychiatry, Psychosomatics, and Psychotherapy, University of RegensburgRegensburg, Germany

**Keywords:** mild cognitive impairment, Alzheimer's disease (AD), ageing, inhibition functions, anti-saccades, fMRI

## Abstract

Performance on tasks that require saccadic inhibition declines with age and altered inhibitory functioning has also been reported in patients with Alzheimer's disease. Although mild cognitive impairment (MCI) is assumed to be a high-risk factor for conversion to AD, little is known about changes in saccadic inhibition and its neural correlates in this condition. Our study determined whether the neural activation associated with saccadic inhibition is altered in persons with amnestic mild cognitive impairment (aMCI). Functional magnetic resonance imaging (fMRI) revealed decreased activation in parietal lobe in healthy elderly persons compared to young persons and decreased activation in frontal eye fields in aMCI patients compared to healthy elderly persons during the execution of anti-saccades. These results illustrate that the decline in inhibitory functions is associated with impaired frontal activation in aMCI. This alteration in function might reflect early manifestations of AD and provide new insights in the neural activation changes that occur in pathological ageing.

## Introduction

Ageing is associated with a decline in cognitive functioning along with structural and functional changes in the brain. A large body of behavioral research on the effect of ageing on human cognition has found that age-related deteriorations are observed across a range of cognitive domains including processing speed, working memory and inhibition (Moscovitch and Winocur, 1992; Braver and Barch, 2002; Anderson et al., 2012; Heilbronner and Münte, 2013). The process of ageing, however, does not inevitably lead to cognitive decline and the extent of the cognitive decline varies in different individuals across various tasks (Rapp and Amaral, 1992; McDowd, 1997; Friedman et al., 2008; Park and Reuter-Lorenz, 2009). Thus, more research is required to understand neurocognitive changes related to human ageing. A major challenge for ageing researchers is to acquire a better distinction between normal and pathological changes with age.

A transitional state between normal and pathologic ageing that is associated with an increased risk of progression to AD is defined as mild cognitive impairment (MCI) (Petersen, 2004). Based on the results of numerous studies on pathological ageing, it is clear that there is no single framework that describes the neuropathology of MCI (Stephan et al., 2012a,b). The complexity and heterogeneity of this condition is reflected in the considerable variability in the definition, characterization and application of the MCI diagnosis in the clinical practice (for the review see: Stephan et al., 2012a). It is widely accepted, however, that there are many subtypes of MCI. According to Petersen (2004), amnestic (aMCI) or non-amnestic MCI (naMCI) should be differentiated from each other. This classification depends on whether memory decline is exhibited in MCI patients. Individuals with intact memory abilities are diagnosed with naMCI as opposed to the individuals with memory impairment diagnosed as aMCI (Petersen, 2003, 2007). It has been suggested that the various MCI subtypes have different etiologies and outcomes (Sachdev et al., 2012). Therefore, individuals diagnosed with aMCI are thought to be more likely to progress to AD (Hunderfund et al., 2006; Petersen and Jack, 2009). In addition to episodic and semantic memory deficits in aMCI patients (Fox et al., 1998; Petersen, 2004; Chertkow et al., 2008), several recent studies suggest that deterioration of executive functions is associated with the conversion to AD (Greenwood, 2000; Rozzini et al., 2007; Iachini et al., 2009; Alichniewicz et al., 2012). Furthermore, there is some evidence that individuals demonstrate deficits in executive functions 2–3 years before diagnosis of AD (Johnson et al., 2009).

Studies on changes in cognition with age showed that while excitatory aspects of attention (like directed visual attention) are preserved in elderly persons, inhibitory processes decline with age (Hasher and Zacks, 1988; Sweeney et al., 2001; Crawford et al., 2005; Peltsch et al., 2011). Age-related inhibitory impairment, however, is restricted to specific inhibitory functions associated with prepotent response inhibition (Butler et al., 1999; Friedman and Miyake, 2004; Mirsky et al., 2011). According to Friedman and Miyake (2004), inhibition of prepotent responses is an ability to deliberately suppress an automatic and dominant action or to ignore no longer relevant contents when necessary. The inhibition of prepotent responses reveals resistance to preseverate and to exhibit controlled and intended reaction (Miyake et al., 2000). As postulated by Miyake et al. (2000), the tasks used to assess this function are the Stroop task (Stroop, 1935), the antisaccade task (Hallett, 1978), and the stop-signal task (Logan, 1994) as they all require the participants to intentionally stop an automatic response. Recent studies showed that performance on oculomotor tasks declines with age (Sweeney et al., 2001; Raemaekers et al., 2006; Peltsch et al., 2011). Elderly adults demonstrate reduced ability to voluntary inhibit saccadic response, as assessed by anti-saccade tasks, and exhibit significantly increased onset latencies of correct anti-saccades compared to young adults (Olincy et al., 1997; Butler et al., 1999; Nieuwenhuis et al., 2000; Crawford et al., 2005; Peltsch et al., 2011). In contrast, their performance on automatic saccadic initiation, as assessed by reflexive pro-saccadic tasks, does not deteriorate in healthy ageing. Older adults, however, need significantly more time to generate pro-saccades accurately (Sweeney et al., 2001; Peltsch et al., 2011).

Altered inhibitory functioning has also been reported in dementia of Alzheimer's type (Currie et al., 1991; Crawford et al., 2005; Mosimann et al., 2005). In a review, Amieva et al. (2004) suggests that the deterioration of inhibitory functions in Alzheimer's disease (AD) may not be a general deficit. In particular, patients with AD demonstrated impairments on tasks requiring controlled inhibitory processes, such as the Stroop task (Fisher et al., 1990; Amieva et al., 2002; Belleville et al., 2006) and the anti-saccade task (Fletcher and Sharpe, 1986; Kaufman et al., 2010, 2012), whilst there is no effect of AD on the performance on tasks requiring more automatic inhibition, such as inhibition of return (Langley and Madden, 2000). The inability of persons with AD to perform anti-saccades may be associated with substantial neural degeneration in the frontal cortex (Fletcher and Sharpe, 1986; Crawford et al., 2005; Kaufman et al., 2010). Furthermore, the literature shows that the anti-saccade task can also be used to distinguish between different types of dementia and other forms of degenerative disorders (Boxer et al., 2006; Garbutt et al., 2008; Lagun et al., 2011).

Despite the fact that there is clear evidence for inhibition deficits in AD, the extent of the deterioration of inhibition has rarely been studied in patients with MCI. Furthermore, there is some inconsistency in the literature with respect to the role of response inhibition in this disorder. While some authors point out that patients with aMCI demonstrate an impairment of inhibitory functions, as assessed by Stroop task (Kramer et al., 2006; Traykov et al., 2007; Bélanger et al., 2010), others argue that MCI has no effect on the performance on this task (Zhang et al., 2007). Moreover, patients with MCI show deterioration of the semantic inhibition of a prepotent response and impairment of inhibition functions may have predictive value with regard to a probable disease progression to AD (Bélanger and Belleville, 2009). On the neural level, increased activity in the dorsal anterior cingulate, bilateral middle and inferior frontal gyri, bilateral inferior parietal lobule, and the bilateral insula was evident in relation to the Stroop task reported in patients with MCI compared to young persons (Li et al., 2009). Crutcher et al. (2009) demonstrated that an oculomotor version of the visual paired-comparison task can be used for assessing normal and impaired recognition memory. Furthermore, Lagun et al. (2011) suggest that machine-learning techniques using the results of a visual paired-comparison task can be used to distinguish MCI from cognitive intact elderly persons. Therefore, there is some suggestions that eye movement functions are impaired in MCI.

To our knowledge, there is no study investigating the neural correlates of oculomotor functions in aMCI. The ability to voluntarily suppress an automatic response in favor of performing an alternative behavior is considered to be one of the crucial executive functions required in everyday life (Munoz and Everling, 2004). Since deterioration of this ability has been found in AD, we hypothesized that neural activation associated with oculomotor performance in anti-saccade tasks should be reduced in persons with aMCI, whereas neural activation for pro-saccades should be unaffected. Specifically, we hypothesize that patients with amnestic MCI will exhibit more errors on the anti-saccade task, while demonstrating reduced activation in saccade-controlling regions in prefrontal and parietal cortex.

## Methods

### Participants

In order to investigate the effects of normal and pathological ageing on cognition and brain activation, three subject groups were assessed: aMCI patients [*n* = 23, mean age = 60.3 (9.3) years], healthy elderly controls [*n* = 19, mean age = 58.8 (7.4) years] and healthy young persons [*n* = 13, mean age = 24.6 (2.1) years]. Participants with MCI were recruited from the Memory Clinic of the Department of Psychiatry, Psychosomatics and Psychotherapy of the University of Regensburg or via advertising in print media seeking healthy adults over 50 years of age who would like to participate in an fMRI-study. In order to exclude possible presence of any other neurological, psychiatric, or systemic condition among both groups, which may lead to alterations in cognitive status, subjects underwent a two-stage screening process to identify those with probable MCI. The first stage consisted of a structured telephone interview assessing evidence of subjective and informant-corroborated reports of memory problems and the presence of any exclusion criteria, including: history of significant medical, neurological, or psychiatric condition, history of major risk factors for vascular disease, history of alcohol and nicotine abuse or sensory impairment (visual acuity), use of psychoactive medication and any aspect that would disqualify a person to enter the MRI scanner (e.g., cardiac pacemaker). The second stage of the screening procedure involved a standardized diagnostic assessment of the cognitive, neuropsychological, and psychological state of the participants. The healthy control group consisted of adults that were recruited from the community via the same ad in the local media. Healthy elderly adults underwent the same clinical and neuropsychological procedure as aMCI patients to ensure that they did not demonstrate any cognitive complaints and did not have any medical history of significance. Finally, young participants were recruited from the community and via word-of-mouth communication. They were examined in the same way as the other two groups of participants, with the exclusion of the test batteries for clinical diagnosis of MCI.

Persons with MCI met the revised criteria for amnestic MCI as proposed by Artero et al. (2006) that included: evidence of decline in memory and other cognitive functions that is, however, insufficient to meet the Diagnostic and Statistical Manual for Mental Disorders–Fourth Edition (DSM–IV) criteria for dementia of the Alzheimer type (American Psychiatric Association, 1994). Neuropsychological assessment consisted of neuropsychological test battery contained in the Consortium to Establish a Registry for Alzheimer's Disease (CERAD+, German version; Memory Clinic Basel, 2005). The critical range of the CERAD was a z-score below −1.5 standard deviations (SD) but not less than −3 SD in at least one CERAD memory subtest for the MCI group and *z*-scores of at least above −1.5 SD in all CERAD subtests for the healthy control subjects. Memory subtests in CERAD+ critical for the diagnosis included: Wordlist Delayed Recall and Savings, TMT-A, Recall of Constructional Praxis, Constructional Praxis: Savings (see Table 1).

**Table 1:** **Description of demographic data and results of neuropsychological testing in amnestic mild cognitive impairment (aMCI) and healthy age-matched control groups**.

**Parameters**	**Healthy elderly**	**aMCI**	***T* (*df* = 40)**	***p***
**DEMOGRAPHY**				
*N*	19	23		
Age (years)	58.84 (7.41)	60.30 (9.31)	0.55	0.582
Gender (M/F)	8/11	5/18	2.02	0.192
Education (years)	14.74 (2.26)	13.09 (3.26)	−1.87	0.069
Mini-mental state exam	29.32 (0.82)	28.91 (1.13)	−1.30	0.201
**CLINICAL CHARACTERISTICS^a^**				
Verbal fluency	29.53 (6.15)	22.61 (5.70)	−**3.78**	**0.001**
Boston naming test	14.74 (0.45)	14.04 (1.11)	−**2.74**	**0.010**
Word list learning	22.58 (2.78)	18.70 (2.99)	−**4.33**	**0.000**
Word list recall	8.79 (1.08)	5.87 (1.71)	−**6.43**	**0.000**
Word list: savings	0.97 (0.09)	0.78 (0.22)	−**3.75**	**0.001**
Word list intrusions	0.16 (0.50)	0.65 (0.98)	**2.10**	**0.043**
Word list recognition	20.00 (0.00)	19.61 (0.66)	−**2.86**	**0.009**
Phonematic fluency	18.68 (3.43)	13.65 (5.01)	−**3.72**	**0.001**
TMT-A	30.67 (7.81)	45.39 (20.41)	**2.89**	**0.006**
TMT-B	69.33 (26.42)	84.83 (35.43)	1.55	0.130
Constructional praxis	10.95 (0.23)	10.74 (0.69)	−1.36	0.184
Recall of constructional praxis	10.58 (0.84)	8.48 (1.93)	−**4.72**	**0.000**
Constructional praxis: savings	0.96 (0.06)	0.79 (0.17)	−**4.50**	**0.000**

Statistical tests of possible group differences (T-values and p-values) are presented.

aMCI, amnestic mild cognitive impairment; denoted are mean values, standard deviations (in brackets), T-, degrees of freedom (df) and p-values.

a Clinical characteristics are mean raw-scores from subtests of the neuropsychological test battery contained in the Consortium to Establish a Registry for Alzheimer's Disease Plus (CERAD-Plus).

Significant values (p < 0.05) are marked in bold font.

All participants were able to understand, and follow verbal instructions, and concentrate on the task for the duration of the experiment. They all were right-handed (according to the Handedness Inventory of Raczkowski et al., 1974), and had normal or corrected-to-normal vision using their own contact lenses or MR-safe refractive corrections provided by us.

### Study design

#### Procedure

The procedures used in this study were approved by the Ethical Committee of the University of Regensburg. In accordance with the requirements of the Code of Ethical Principles for Medical Research Involving Human Subjects of the World Medical Association (Declaration of Helsinki), all participants provided written informed consent prior to the commencement of the study.

#### Pro-saccade and anti-saccade task

In order to investigate the effect of normal and pathological ageing on prepotent inhibition, the pro- and anti-saccades paradigm was employed. During each trial, a fixation cross (0°) appeared in the center of the computer display for a variable amount of time 5000, 5100, or 5200 ms (durations jittered with 100 ms increments). A target stimulus was presented on one of four different peripheral positions along the horizontal midline (±5 and 15°) for 200 ms. The location of the target was randomized and counterbalanced so that participants were unable to predict at which position the target would be presented next. The saccade target was presented at one of the possible locations for 200 ms. Then, a visual feedback appeared at the eccentric location of the pro-saccade target or anti-saccade location (a tiny green dot), visible only when the fovea was near the target location. The distractor locations were baited with a tiny red dot. On error trials, the red dot was visible to the participant, signaling that they executed a saccade to the wrong location. After this, the feedback dot was extinguished to be replaced immediately with the centrally located fixation dot, inducing a recentering saccade. Feedback was given on all trials in both training and fMRI sessions. After 1800 ms of central fixation, another trial began. Each task was performed 8 times during 5 blocks for pro- and anti-saccade tasks each, with a total number of 80 trials. On pro-saccade trials the central fixation dot turned green 500 ms prior to fixation offset, while on anti-saccade trials it turned red (see e.g., Connolly et al., 2002; Cornelissen et al., 2002). Also, a visual instruction (“look toward the target” vs. “look away from the target” (“Hinschauen” and “Wegschauen” in German, respectively) was presented for 5000 ms before each block of trials in each task. Participants were instructed to look toward the peripheral target as fast as they could during the “green” trials and to look as fast as possible to the mirror image location of the peripheral target during the “red” trials. During fixation epochs, participants were asked to keep their eyes on the central target. Approximately one week before the fMRI scan, all participants performed a pro-saccade and anti-saccade training experiment. We acquired 40 trials of pro-saccades and 40 trials of anti-saccades, in a similar blocked fashion as to be applied in the scanner, using the video eyetracker. Additionally, all participants were shown 8 trials of each task prior to the fMRI scan on the day of examination to refresh their memory on the task specifics. Feedback was also presented during these training trials.

#### Stimulus presentation and eye-tracking

For stimuli presentation we used Presentation 9.9 (Neurobehavioral Systems Inc., Albany, California, USA) on a standard PC, equipped with a standard graphics card and back projected via an LCD video projector (JVC, DLA-G20, Yokohama, Japan) onto a translucent circular screen (approximately 30 degrees visual angle in diameter), placed inside the scanner bore at 62 cm from the observer. The projector was running at 72 Hz with a resolution of 800 × 600 and a color resolution of 3 × 8 bit (RBG). During training outside the MR-scanner we used High Speed Video Eye-Tracker Toolbox™ (Cambridge, http://www.crsltd.com/catalog/eyetracker-250/index.html), where the participant was seated upright with their chin resting on a chinrest. During the fMRI scan, subjects viewed the stimuli through a mirror located above their eyes. Eye movements were recorded using the MR-Eyetracker (Cambridge Research Systems, Ltd), a fiberoptic limbus-tracking device positioned on the headcoil to monitor task performance. Owing to problems related to signal-to-noise in the eye-tracking data collected in the scanner, we were only able to verify task compliance “on-line” and could not conduct a off-line analysis of the eye-movement data. The results reported in Table 3 (see below) were collected during the training period outside of the scanner using the video eyetracker.

#### Magnetic resonance imaging

Functional magnetic resonance imaging (fMRI) was conducted on a 3-Tesla head scanner (Siemens Allegra, Erlangen, Germany). A localizer scan for placing the volume of interest was first acquired. In order to obtain a 3D anatomical model of the brain scan, high-resolution, sagittal T1-weighted images were acquired by using a magnetization-prepared gradient echo (MP-RAGE) sequence. The anatomical data set consisted of 160 sagittal slices, FoV 256 mm, slice thickness 1.00 mm, TR 2250 ms, TE 2.6 ms, flip angle 90°. For functional analysis a total of 610 functional volumes were acquired. Each functional scan contained 34 3-mm slices, positioned oblique to the axial plane using a T2^*^-weighted EPI sequence (*TR* = 2.0 s, *TE* = 30 ms, 3 × 3 × 3 mm^3^ voxel size, flip angle 90°).

### Data analysis

The evaluation of the eye movements was conducted manually using Matlab. Owing to the limitations on the signal-to-noise of the in-scanner eyetracking data, we base our analysis of the behavioral data on the video eyetracking data acquired during the training session outside of the scanner. We analyzed the eye trajectories offline and evaluated the task performance of the subjects. For both tasks, hit rates of the correct eye movements and reaction times were calculated. Accuracy rates among the groups exceeded chance performance in every group. A saccadic response was considered correct when the eye-movement direction was correct and the saccade landing point was near (±2°) the peripheral visual target location. Reflexive pro-saccades that were not executed toward the mirror image location of the peripheral target were classified as errors in the anti-saccade task. Saccadic reaction time was calculated as the time from stimulus onset to saccadic onset. In the anti-saccade task, only data from trials where the saccades directed gaze toward the mirror location of the visual stimulus were scored as correct. For both conditions, saccades with reaction time less than 100 ms were classified as anticipatory and thus excluded from further analysis (0.5% of all saccades). The groups did not differ with respect to frequency of anticipatory saccades. Group comparisons of the behavioral data were conducted using *T*-test for independent samples in SPSS 18.0 (SPSS Inc., Chicago, Illinois). All tests were two-tailed, with a value of *p* < 0.05 to determine statistical significance. During fMRI-scanning, the eye-position trace was observed to confirm in all participants that they performed the task as instructed. The experimenter noted any lapses on the part of the participant, as well as excessive eye blinks. We had no indication that any of the participants did not understand the tasks, nor did we have any evidence that any of the participants did not perform the task while in the scanner.

The functional MRI data were preprocessed and analysed using Statistical Parametric Mapping 5 (SPM, Wellcome Trust Centre for Neuroimaging, University College London, UK, http://www.fil.ion.ucl.ac.uk/spm). To correct for differences in image acquisition time between slices, *slice timing* correction was conducted. The movement artifacts in the time-series of images were removed using a least squares approach and a six-parameter (rigid body) spatial transformation. Maximum head movement never exceeded 3 mm and no scans had to be removed due to excessive head motion. The images were realigned to spatially match the first image. The structural image was realigned to a mean image computed from the functional series. All images were then normalized to the Montreal Neurological Institute (MNI-152) space. The realigned and normalized functional series were resampled to 2 × 2 × 2 mm resolution and spatially smoothed with a Gaussian kernel of 8 mm *Full-Width at Half-Maximum* (FWHM).

**Figure 1:**
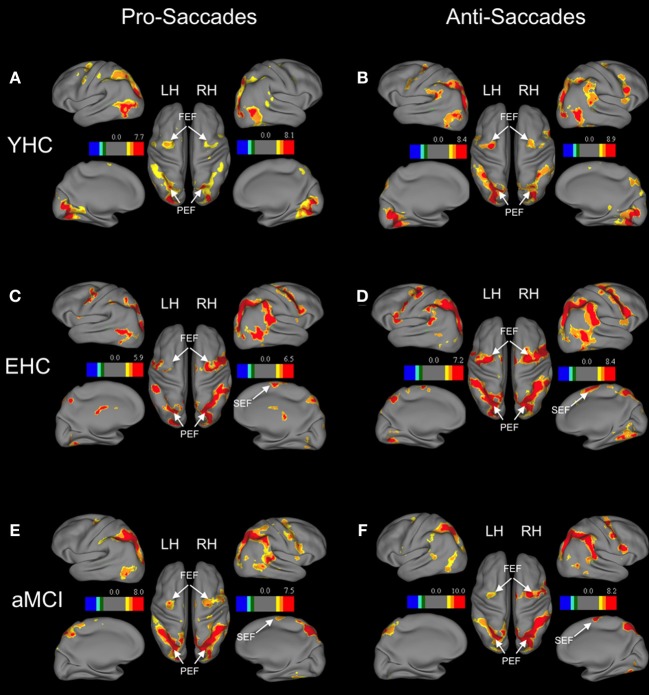
**Brain areas showing significant activation associated with pro-saccades and anti-saccades in all subject groups (contrasts: pro-saccades > baseline, anti-saccades > baseline).** (Panels **A,C,E**) present the results for the pro-saccade condition, whereas (Panels **B,D,F**) display the results for the anti-saccade condition. Abbreviations: YHC, young healthy controls; EHC, elderly healthy controls; aMCI, patients with mild cognitive impairment; LH, left hemisphere; RH, right hemisphere; FEF, frontal eye fields; SEF, supplementary eye fields; PEF, parietal eye fields. Color insets present the *T*-values for the contrast saccade condition vs. baseline.

For the statistical analysis, the convolution of a canonical hemodynamic response function (HRF) with square temporal onset profiles (boxcars), representing the onsets of the relevant experimental conditions (the stimulus onset events for pro-saccades, anti-saccades, and fixation), were used to define the regressors, after removing the first three volumes. Except for anticipatory saccades, all trials (correct and incorrect) entered into the analysis. Motion parameters estimated during preprocessing of the functional images were included as additional regressors in the GLM model. Instructions and feedback were modeled as separate events of no interest. Single subject *T*-contrast maps were derived by *t*-statistics utilizing the canonical haemodynamic response function (HRF). In a second level random-effects analysis, between group differences in neural activation associated with the relevant contrasts were assessed using a one-way ANOVA. Within this ANOVA two-sample *t*-tests were computed to analyse effects between young healthy subjects and healthy elderly, as well as between healthy elderly and MCI patients. Clusters of *k* ≥ 10 contiguous voxels large enough to pass a cluster-wise threshold of *p* < 0.001 were considered as significant. Active brain areas were labeled with anatomical loci and Brodmann areas by using the SPM5 extension Wake Forest University (WFU) pick-atlas (Maldjian et al., 2003). The WFU pick-atlas was also used to convert MNI- in Talairach-coordinates. To assess age related changes in brain activation, the regions of interest (ROI) based approach was employed. The analysis was done on subject-specific parameter estimates extracted from the peak voxel within each region of interest using the MarsBAR toolbox in SPM5 (*Mars*eille *B*oîte *À R*égion d'Intérêt; http://marsbar.sourceforge.net/). This strategy allows for an anatomically focused analysis of the role of specific brain areas in saccade and anti-saccade task performance (Connolly et al., 2002; Curtis et al., 2004). ROIs were chosen on the basis of the relevant literature and our *a priori* hypothesis that activation of these regions would be modulated by the age and cognitive status of the participants. The regions associated with pro-saccadic and anti-saccadic eye movements have been well-described in the literature (Connolly et al., 2002; Matsuda et al., 2004; Brown et al., 2006; Parton et al., 2007; Hutton, 2008). Previous studies showed that fronto-parietal network is crucial for the planning and execution of saccadic eye movements (Rivaud et al., 1994; Matsuda et al., 2004; Grosbras et al., 2005). There is some inconsistency; however, whether frontal eye fields (FEF) or intraparietal sulcus (IPS) is more active during anti-saccades compared with pro-saccades (Connolly et al., 2002; Cornelissen et al., 2002; Curtis et al., 2004; Brown et al., 2006; Ettinger et al., 2008). Thus, the sets of regions of interest were defined for prosaccades and anti-saccades that included frontal and parietal regions as well as supplementary eye fields. Therefore, we used a literature-derived anatomical approach to investigate the effect of normal and pathological ageing on saccade-rated activations within frontal, parietal and supplementary eye fields. The ROIs with 5 mm radius (10 mm diameter) are displayed in Table 2 and labeled in Figure 1. First, we defined an anatomical mask (structural ROIs) for each subject based on his or her high-resolution structural scan. Then, the magnitude of activation within each ROI was calculated for each subject as the percent signal change for the relevant conditions over the voxels within the regions of interest (Poldrack, 2007).

**Table 2:** **Anatomical locations of regions of interest in the left and right hemispheres**.

**Cortical region (ROI)**	**Talairach coordinates (in mm)**		
	***x***	***y***	***z***
Frontal eye fields	−32	16	48
	20	8	48
Parietal eye fields	38	−47	48
	−43	−50	52
Supplementary eye fields^*^	17	−17	56

* Activation in supplementary eye fields is pooled over left and right hemispheres.

## Results

### Behavioral data

The analysis of the behavioral performance measured outside of the scanner revealed that the groups did not differ in accuracy on the pro-saccade task (Table 3). In contrast, healthy elderly persons and aMCI-patients exhibited more direction errors while performing anti-saccades. However, the aMCI group did not differ significantly with respect to the proportion of omissions (i.e., proportion of trials where they executed no saccade) compared to the age-matched controls (Table 3). Compared to young persons, healthy elderly persons executed significantly fewer correct anti-saccades and needed significantly more time to perform the task correctly. Furthermore, healthy elderly persons were significantly slower in pro-saccade task in comparison with young persons. Patients with aMCI executed significantly fewer correct anti-saccades compared to elderly healthy persons. The reaction times, however, did not differ significantly in either of tasks between both groups.

**Table 3:** **Performance measured outside of the scanner (proportion correct among executed saccades, proportion of omissions, reaction times in ms) of young persons, healthy elderly and aMCI patients in pro-saccade and anti-saccade task**.

**Parameters**	**Young persons vs. healthy elderly**				**Healthy elderly vs. aMCI**		
	**Young persons**	**Healthy elderly**	***t*(df)**	***p***	**aMCI**	***t*(df)**	***p***
**HIT RATES^a^**							
Pro-saccades	0.99 (0.01)	0.99 (0.01)	0.462 (30)	0.648	0.98 (0.03)	1.100 (40)	0.278
Omissions	0.001 (0.0001)	0.043 (0.208)	−0.956	0.339	0.071 (0.262)	−0.416	0.676
Anti-saccades	0.96 (0.04)	0.74 (0.16)	−**4.865** (30)	0.000^*^	0.57 (0.18)	**3.063** (40)	0.04^*^
Omissions	0.0045 (0.0006)	0.023 (0.104)	−0.1.078	0.281	0.036 (0.089)	−1.496	0.135
**REACTION TIMES (ms)**							
Pro-saccades	249.2 (28.3)	285.6 (53.7)	**2.227** (30)	0.034^*^	306.3 (241.4)	−0.908 (40)	0.370
Antisaccades	338.5 (21.8)	478.3 (119.6)	**4.146** (30)	0.000^**^	509.0 (118.6)	−0.804 (40)	0.427

aMCI, amnestic mild cognitive impairment; n.s., not significant; denoted are mean values, standard deviations (in brackets), Z- and p-values. Levels of significance indicated by askerisks:

* p < 0.05;

** p < 0.01.

a Note that there were 4 possible target positions. Thus, performance by chance would correspond to a hit rate of 25%.

Significant values (p < 0.05) are marked in bold font.

### Functional imaging data

Separate analyses for each group showed increased activation of distributed frontal and parietal regions in both pro-saccades and anti-saccades (Table 4). These areas are known to be involved in the execution of saccadic eye movements.

**Table 4:** **Results of regions of interest analysis in young persons, healthy elderly and aMCI patients (BOLD response as percent signal change within ROI across all trials)**.

**Regions of interest**	**Young persons vs. healthy elderly**				**Healthy elderly vs. aMCI**		
	**Young persons**	**Healthy elderly**	***t*(df)**	***p***	**aMCI**	***t*(df)**	***p***
**PRO-SACCADES**							
**Frontal eye fields**							
Right	0.20 (0.12)	0.21 (0.23)	0.155 (30)	0.878	0.14 (0.13)	1.238 (40)	0.223
Left	0.18 (0.15)	0.13 (0. 16)	−0.827 (30)	0.415	0.11 (0.10)	0.308 (40)	0.759
**Parietal oculomotor area**							
Right	0.40 (0.24)	0.26 (0.20)	−1.670 (30)	0.106	0.23 (0.19)	0.535 (40)	0.596
Left	0. 38 (0.24)	0. 27 (0. 18)	−1.363 (30)	0.183	0.22 (0.21)	0.750 (40)	0.458
**Supplementary eye fields**							
	0.17 (0.18)	0.11 (0.16)	0.827 (30)	0.422	0.19 (0.27)	−1.237 (40)	0.223
**ANTI-SACCADES**							
**Frontal eye fields**							
Right	0.32 (0.17)	0.32 (0. 20)	−0.049 (30)	0.961	0.16 (0.12)	**3.030** (40)	0.004^**^
Left	0.32 (0.19)	0. 25 (0.14)	−1.003 (30)	0.329	0.15 (0.15)	**2.202** (40)	0.033^*^
**Parietal oculomotor area**							
Right	0.57 (0.21)	0. 39 (0.20)	−**2.319** (30)	**0.028**	0.29 (0.21)	1.645 (40)	0.108
Left	0.54 (0.17)	0.37 (0.18)	−**2.510** (30)	0.018^*^	0.28 (0.22)	1.532 (40)	0.133
**Supplementary eye fields**							
	0.20 (0.23)	0.27 (0.28)	−0.633 (30)	0.534	0.23 (0.34)	0.406 (40)	0.687

aMCI, amnestic mild cognitive impairment; denoted are mean values, standard deviations (in brackets), t- and p-values, df: degrees of freedom. Levels of significance indicated by askerisks: ^*^p < 0.05; ^**^p < 0.01.

Significant values (p < 0.05) are marked in bold font.

Region of interest analysis revealed that there are no significant differences in the neural activation between groups while performing pro-saccade task (pro-saccades > fixation of healthy elderly compared to pro-saccades > fixation of aMCI; Table 4). There are, however, significant changes in the activation associated with anti-saccade task with respect to age as well as with the aMCI diagnosis. While healthy elderly showed significantly less activation bilaterally in parietal regions compared to young persons, patients with aMCI exhibited significantly decreased activation bilateral in frontal regions (FEF) in comparison to healthy elderly participants.

## Discussion

The present study aimed to explore the effects of normal and pathological ageing on inhibitory processes underlying the control of reactive saccade execution. In particular, we investigated the behavioral and neural correlates of prepotent response inhibition, assessed by the anti-saccade task, as postulated by Miyake et al. (2000) and Friedman and Miyake (2004). Our findings point to a significant impairment in the ability of aMCI patients to perform the antisaccade task. Furthermore, our findings indicate that this impaired execution of antisaccades is associated with significantly reduced BOLD signal in the frontal eye fields in aMCI patients. Our results are consistent with earlier studies (e.g., Amieva et al., 2004) on the effects of ageing and dementia on saccadic execution.

### Saccadic and anti-saccadic eye movements in normal ageing

In agreement with studies on the decline of inhibitory processes with age (e.g., Hasher and Zacks, 1988; Nieuwenhuis et al., 2000; Sweeney et al., 2001; Raemaekers et al., 2006; Peltsch et al., 2011), our study demonstrates a reduced ability of elderly adults to voluntarily inhibit saccadic responses compared to young adults. Moreover, the onset latencies of pro-saccades and anti-saccades were significantly longer in healthy elderly adults compared to young adults (Table 3). The performance of elderly adults on the automatic saccadic initiation, however, showed no decline compared to the performance of young adults on the pro-saccade task. Such findings have been documented by previous research on pro-saccadic and anti-saccadic eye movements (Olincy et al., 1997; Butler et al., 1999; Klein and Foerster, 2001; Sweeney et al., 2001; Crawford et al., 2005; Peltsch et al., 2011). Moreover, some researchers demonstrated that the poor performance in the anti-saccade task and longer saccadic latencies cannot only be found in elderly persons but also in children aged between 5 and 8 years (Munoz et al., 1998). Thus, these behavioral changes in performance may reflect different stages of normal development as well as degeneration in the nervous system (Munoz et al., 1998).

### Saccadic and anti-saccadic eye movements in pathological ageing

The present study revealed impaired inhibitory functions in aMCI patients, as assessed by the anti-saccade task. Our results agree with those of previous studies that show altered inhibitory functions on Stroop task in aMCI patients (Traykov et al., 2007; Bélanger et al., 2010). Controversially, Zhang et al. (2007) failed to find significant differences in performance on Stroop task in MCI. These contrasting findings point to the heterogeneity underlying MCI (Nordlund, 2005). Furthermore, deterioration of controlled inhibition processes and preserved ability to execute pro-saccades has been widely reported in patients with AD (e.g., Currie et al., 1991; Amieva et al., 2002; Crawford et al., 2005; Mosimann et al., 2005; Collette et al., 2009). Moreover, there is evidence in the literature that persons diagnosed with AD demonstrate different performance patterns in oculomotor tasks compared to patients with Parkinson's disease with dementia, dementia with Lewy bodies (Mosimann et al., 2005), frontotemporal lobar degeneration, corticobasal syndrome and progressive supranuclear palsy (Garbutt et al., 2008). Thus, the anti-saccade task can also be used to distinguish between different types of dementia and other forms of degenerative disorders. Given the fact that persons with aMCI are likely to develop AD (Petersen and Jack, 2009), our findings illustrate that assessment of the prepotent inhibitory functions in aMCI might be sensitive to early manifestations of AD.

### Neural correlates of inhibitory processes in normal ageing

The analysis of neural activation associated with saccadic inhibition showed that all groups found significant activations in regions that are thought to underlie the execution of pro- and anti-saccades (Pierrot-Deseilligny et al., 1991; Matsuda et al., 2004; Ford et al., 2005; Parton et al., 2007). A ROI analysis of the anti-saccade task revealed that healthy elderly adults show significantly less activation bilaterally in the inferior parietal lobe compared to young adults. This change in neural activation was accompanied by a significant reduction in performance accuracy and longer onset latencies. However, neither parietal nor frontal regions showed significant changes in activation during the pro-saccade task, even though elderly healthy adults needed significantly more time to perform as accurately as young adults. In contrast, Raemaekers et al. (2006) reported no significant effect of age on the neural activation with respect to saccadic inhibition but lower activation in older adults compared to young and middle-aged adults who executed pro-saccades. Nevertheless, no significant difference in performance on either task was found between groups (Raemaekers et al., 2006). Nelles et al. (2009) showed an age-dependent increase in activation in a pro-saccade task in bilateral parietal eye fields, the right frontal eye field, as well as in the right extrastriate cortex. However, the previous study did not report any findings on the task performance of the individuals. The discrepancy between studies of Raemaekers et al. (2006); Nelles et al. (2009) and our study may be explained with increased interindividual variability in performance in cognitive tasks, which has been widely reported in elderly adults (for a review: Hedden and Gabrieli, 2004; Bishop et al., 2010). Also, the number of participants in both studies is lower compared to our study: Raemaekers et al. (2006) investigated three groups with a total number of 36 persons whereas the subject population in the study of Nelles et al. (2009) contained in total 22 persons. Furthermore, the present findings are consistent with those of previous studies illustrating effects of normal ageing on performance in oculomotor tasks (e.g., Fletcher and Sharpe, 1986; Mulligan et al., 1996). Hence, our findings extend these results and show an association between decreased activation in the parietal lobe in elderly adults and a decline in response inhibition.

Previous fMRI studies stressed the role of parietal areas in the execution of anti-saccades (Kimmig et al., 2001; Curtis and D'Esposito, 2003; Brown et al., 2006). More recently, Sharpe et al. (2011) showed that impaired suppression of reflexive saccades and generation of anti-saccades could be attributed to a partial disconnection of the parietal lobe from frontal lobe ocular motor areas. Given that reduction in fractional anisotropy of frontal and parietal white matter increases with age (Abe et al., 2002; Grieve et al., 2007), a decrease in parietal activation in elderly adults may result as a change in functional connectivity between these regions as seen in our study. Moreover, decreased activation in parietal regions together with reduced performance was observed in elderly adults in a Stroop task (Milham et al., 2002). In another study, right prefrontal and parietal regions were more activated in elderly adults compared to young persons during “successful inhibition” assessed with the Stroop interference paradigm (Nielson et al., 2002). This activation patterns can be explained by theoretical models of ageing (Cabeza et al., 2004; Grady et al., 2008; Reuter-Lorenz and Park, 2010) postulating that increases (or decreases) in activation refer to neural resources that are engaged (or disengaged) in healthy elderly to cope with cognitive challenges. Consistent with those models, neuroimaging findings indicate that in cases of limited cognitive decline with age, an over-activation is observed in regions important for the cognitive tasks in question (Cabeza et al., 2004). However, when cognitive impairment is more advanced, both decreased neural activity and poor performance on behavioral tasks have been reported in elderly adults (Persson and Nyberg, 2006). Hence, the failure to differentially activate the parietal regions may indicate neural inefficiency in elderly adults as seen in our study, accompanied by a decline in the ability to perform the demanding anti-saccade task requiring saccadic inhibition. Owing to the limitations with respect to the signal-to-noise of the in-scanner eye-tracking data, we only used the eye-movement data online to confirm that the participants performed the task. The non-linear changes in the eye-tracking signal and the low signal-to noise level, however, made a detailed off-line analysis unfeasible. Thus, we were not able to conduct a detailed off-line analysis of the eye-movement data collected in the scanner.

### Neural correlates of inhibitory processes in pathological ageing

Another important focus of our study was directed to the effects of pathological ageing on saccadic inhibition. To our knowledge, this is the first study that investigated anti-saccades and their neural correlates in aMCI. We found that while there is no significant difference in activation related to the pro-saccade task between aMCI patients and elderly adults, aMCI patients show significantly reduced activation in FEF bilaterally during the execution of anti-saccades. Given the fact that aMCI patients demonstrate a significant decline in the ability to correctly execute anti-saccades as compared to healthy elderly, these findings provide new evidence for an association between neural and cognitive deficits in pathological ageing. The reduced activation we observed in the aMCI group compared to the age-matched controls cannot be explained by a larger proportion of omissions in the anti-saccade task. At least for the measurements made outside of the scanner the two groups did not differ with respect to the proportion of omissions during either anti- or pro-saccade tasks (Table 3).

Similar to the fMRI findings on the control of pro-saccades and anti-saccades in aMCI, there is only a limited number of studies that examine the effect of pathological ageing on neural activation associated with oculomotor tasks. Only one other study has investigated brain activation patterns during visually guided saccade paradigm in persons diagnosed with probable AD compared to healthy volunteers. Thulborn et al. (2000) observed left-dominant parietal and prefrontal cortical activation in most patients with probable AD while executing pro-saccades. An explanation for this contrasting result could be the difference between clinically diagnosed probable AD and MCI, which both are pathologically heterogeneous disorders that vary in presentation, onset, or clinical course (Schneider et al., 2009). Furthermore, the inability of persons with AD to perform anti-saccades has been attributed to frontal lobe degeneration (Fletcher and Sharpe, 1986). Also the level of the performance on the Stroop task in patients with AD depends on the functional integrity of the prefrontal cortices (Yun et al., 2011). In a study investigating white matter tracts in MCI, authors showed that tract degeneration in frontal and cingulate regions as well as cortical thinning in caudal middle frontal region are associated with impairment in executive functions (Grambaite et al., 2011). Hence, the decrease in neural activation in FEF during anti-saccade task in patients with aMCI may be an analogous effect to the one seen in patients with AD. Furthermore, findings from our previous study demonstrated no significant differences in gray matter (VBM) between healthy elderly and persons with MCI (Alichniewicz et al., 2012). Moreover, the fact that the BOLD response is normal for pro-saccades in MCI speaks against an anatomical change in the gray matter in these patients and emphasizes the pathological functional disabilities. Therefore, our findings illustrate that the decline in functions for response inhibition accompanied by disrupted frontal activation in aMCI might be sensitive to early manifestations of AD.

### Shortcoming of current study and motivation for future research

Our study characterizes the extent of oculomotor system impairment and altered neural activation in brain areas associated with response inhibition in normal and pathological ageing. The present study relies on eye-tracking performance outside the scanner for comparing the groups. Owing to problems related to signal-to-noise in the eye-tracking data collected in the scanner, we could only verify task compliance “on-line” and were unable to conduct any detailed off-line analysis of the eye-movement data that could have been used to determine error-related processing, perform a parametric analysis related to saccadic reaction times, or determine the role of correction saccades in the pattern of activation found in the aMCI group. Such interesting and relevant analyses are therefore left to future studies.

Moreover, randomized pro- and anti-saccade trial sequences would avoid habituation or adaptation effects associated with block presentation of the different saccade types. However, within each block saccade direction and amplitude were randomized to minimize such adaptation effects. The results of many previous studies suggest that a number of different processes underlie a cognitive decline in AD and MCI (for a review: Stephan et al., 2012a). Thus, we believe that determining the factors that account for behavioral and functional changes reported in this study requires further research.

Furthermore, studies investigating functional connectivity suggest that spontaneous fluctuations in the BOLD signal may contribute to the variability in evoked signals and performance levels (Fox et al., 2006; Fox and Raichle, 2007). Resting-state functional connectivity between the FEF and prefrontal as well as intraparietal cortex exhibits a remarkable similarity across macaque and humans (Hutchison et al., 2012). Also, the extent of task-related reduction in neural activity in default mode regions was shown to be less pronounced in older than in young adults (Lustig et al., 2003; Grady et al., 2006), especially with increasing cognitive task demands (Persson et al., 2007). Since we used a general linear model analysis that is not as sensitive to temporal change in activation and deactivation patterns in dementia as are exploratory data analysis techniques, such as independent component analysis (ICA) (Rombouts et al., 2009), we cannot eliminate the possibility that we may not have been able to detect additional unknown group differences in our neuroimaging data. Hence, important implications regarding the role of the age reductions in the ability to suppress default mode activity in elderly adults during demanding inhibition tasks such as anti-saccade task need to be investigated.

## Conclusions

The aim of this study was to further our understanding of cognitive and neural changes in normal and pathological ageing with respect to inhibitory functions, in particular inhibition of prepotent responses measured in anti-saccade tasks. The present study confirms and extends existing results showing that healthy ageing leads to reduced response inhibition and decreased neural activation in the parietal lobe. Furthermore, our study provides new insights in the neural changes associated with the execution of anti-saccades that occur in pathological ageing. Our functional MRI data revealed that patients with aMCI show reduced activation in frontal eye fields together with reduced ability to execute anti-saccades. A reduced ability to voluntarily suppress an automatic response in favor of performing an alternative behavior is considered to be one of the crucial executive functions required in everyday life (Munoz and Everling, 2004). An analysis of the error-related activations on erroneous anti-saccade trials would have been informative. Thus, future work should address whether alteration of neural activation associated with oculomotor performance found in aMCI can help to detect persons at risk of developing Alzheimer's disease.

### Conflict of interest statement

The authors declare that the research was conducted in the absence of any commercial or financial relationships that could be construed as a potential conflict of interest.
